# Human Papillomavirus Positivity in the Anal Canal in HIV-Infected and HIV-Uninfected Men Who Have Anal Sex with Men in Guangzhou, China: Implication for Anal Exams and Early Vaccination

**DOI:** 10.1155/2017/2641259

**Published:** 2017-01-04

**Authors:** Xuqi Ren, Wujian Ke, Heping Zheng, Ligang Yang, Shujie Huang, Xiaolin Qin, Bin Yang, Huachun Zou

**Affiliations:** ^1^Guangdong Provincial Center for Skin Disease & STI Control, Guangzhou, Guangdong 510091, China; ^2^School of Public Health, Sun Yat-sen University, Guangzhou, Guangdong 510085, China; ^3^Kirby Institute, The University of New South Wales, Sydney, NSW 2052, Australia

## Abstract

*Background.* The epidemiology of HPV in men who have sex with men (MSM) in Guangzhou, China, had not been reported previously.* Methods*. HIV-infected and HIV-uninfected MSM were recruited from a Guangzhou-based MSM clinic in 2013. Sociodemographic characteristics and sexual behaviors were collected. An anal cytological sample was taken for HPV testing.* Results.* We recruited 79 HIV-infected and 85 HIV-uninfected MSM. The median age was 26 years in both groups. The positivities of anal HPV of any type (81.0% versus 48.2%), any high risk type (50.6% versus 27.1%), any low risk type (55.7% versus 31.8%), and any 9-valent vaccine type (74.7% versus 36.5%) were all significantly higher among HIV-infected compared to that among HIV-negative MSM (*p* for all < 0.05). The great majority of HPV-infected MSM were infected with 9-valent vaccine types (59 out of 64 HIV-infected and 31 out of 41 HIV-uninfected). Anal bacterial infections were associated with higher anal HPV positivity and greater number of anal HPV types.* Conclusion.* Sexually active MSM in Guangzhou, especially those infected with HIV, had high and multiple HPV detections. The majority of these cases were potentially preventable by HPV vaccine. Regular anal exams and early HPV vaccination are warranted in this population.

## 1. Introduction

Human papillomavirus (HPV) is one of the most common sexually transmitted infections (STIs) seen in the anogenital area, with high prevalence and incidence among men who have sex with men (MSM) [1–3]. A meta-analysis of global data on HPV in MSM found prevalence of anal HPV to be 93% in HIV-positive MSM and 64% in HIV-negative MSM and these rates did not decrease with increasing age [4]. HPV can cause symptoms such as anogenital warts and intraepithelial neoplasia and squamous-cell carcinoma in the anal canal [5]. These symptoms cause high psychological and financial burdens in the MSM population. Risk factors, including unprotected anal intercourse (UAI) and multiple sex partners, render MSM vulnerable to anal HPV infection, especially among those who are infected with HIV [6, 7]. A meta-analysis found significant difference in the incidence of anal cancer between HIV-infected and HIV-uninfected MSM: 46 versus 5 per 100,000 person-years [4].

Studies in China reported high prevalence of anal HPV among MSM irrespective of HIV status: 65.3–71.4% in Beijing [8–10], 73.0% in Taiyuan [11], 52.7% in Xi'an [12], 68.7% in Chengdu [11], and 36.4% in Shenzhen [13]. Among HIV-infected MSM the HPV prevalence was even higher, ranging from 71.4% in Shenzhen [13] to 99.0% in Xi'an [12]. Guangzhou is the largest city in southern China with a population of over 13 million in 2014 [14]. It was estimated that there were around 25,000 MSM living in Guangzhou [15]. However until now there had been no study that reported on HPV infection among MSM in this city. Our study aimed to clarify the severity of and factors associated with HPV infection among MSM in Guangzhou, comparing HIV-infected to HIV-uninfected ones.

## 2. Methods

### 2.1. Study Population

This study was conducted between January and October 2013 at the STD Clinic of the Lingnan Fellows Health Support Center, the largest MSM health support NGO group in Guangzhou. We recruited HIV-infected MSM from those who attended the clinic for periodical CD4+ T cell count test and HIV-uninfected MSM from those who attended the clinic for HIV testing and who tested negative. Eligible participants were males aged 18 years or older who had had receptive anal sex with another man in the past 12 months. The study was reviewed and monitored by the Ethics Committees of Guangdong Provincial Dermatology Hospital, Guangzhou, China. Written informed consent was obtained from all participants before data and sample collection.

### 2.2. Data Collection

A self-administered questionnaire was collected. The questionnaire included information on sociodemographic characteristics (e.g., age, ethnicity, education, marital status, and career), sexual behaviors (e.g., gender of first sex partner, heterosexual and homosexual behaviors, and condom use in the past three months), knowledge about sexual partner's STD status, experience of commercial sex, and alcohol use. A unique code was used to link questionnaire to specimens of a participant. Personal contact information, which was blinded to researchers, was kept by the STD Clinic of Lingnan Fellows Health Support Center for test results feedback and data validation.

### 2.3. Sample Processing

A blood sample was collected from HIV-negative/unknown subjects for HIV serological assessment. The HIV serologic status was determined by enzyme-linked immunoassay (ELISA, Wantai Biological Medicine Company, Beijing, China). Positive samples by ELISA were further confirmed by using the HIV-1/2 Western blot assay (HIV Blot 2.2 WB; Genelabs Diagnostics, Singapore). HIV serology was performed at the Guangzhou Center for Disease Control. An anal swab sample was taken to test for chlamydia, gonorrhea, and* Mycoplasma genitalium* using nucleic acid amplification test (DaAn Gene Co., Guangzhou, China) as previously described [16].

A clinician checked warts in the anal canal with the help of an anal speculum, after the collection of a cytological sample. A cytological sample was obtained around the dentate line in the anal canal using a small soft cytology brush (HybriBio, Chaozhou, China). The swab sample was coded and stored at −70°C before further processing. HPV DNA was extracted by Cell Lysate Kit (HybrioBio, Chaozhou, China) according to the manufacturer's instructions. HPV DNA was amplified by using MY09/11 primers [17] and the amplified fragment was about 450 bp. The DNA sequences within the L1 region of HPV were used to detect generic HPV DNA. Amplification was carried out in 50 *μ*L of reaction mixture (1x GoTaq PCR buffer (Promega, USA), 0.2 mM deoxynucleotide triphosphates (dNTPs, Thermo, USA), 1.5 mM MgCl_2_ (Sigma-Aldrich, USA), 1 *μ*mole of each primer, 1.25 units (U) of GoTaq DNA Polymerase (Promega, USA) and 2 *μ*L of DNA sample). The amplified condition was 95°C for 2 min of denaturation and then 40 cycles of amplification: 95°C for 1 min of denaturation, 55°C for 1 min of annealing, 72°C for 1 min of extension, and final extension at 72°C for 10 min by using the GeneAmp RCR System 9600 (Perkin Elmer, United States). PCR products were analyzed on 2.0% agarose gel with GeneGreen Nucleic Acid Dye (TianGen, China), visualized by the Tanon 5299 Multi (Ewell, China), and their molecular weights were determined by comparison with a DL 1000-bp DNA ladder (TaKaRa, Japan). All samples were tested by PCR methods three single times and confirmed by Sangon Biotech, China.

HPV genotype screening was conducted using (1) sequencing tests: HPV positive PCR products were purified by PureLink Quick Gel Extraction Kit (Invitrogen, USA) and the sequencing services were purchased from Sangon Biotech, China. The assembled sequences were submitted to BLAST alignment (https://www.ncbi.nlm.nih.gov/blast), against sequences available in GenBank; (2) HPV reverse dot blot genotyping: HPV genotyping was detected according to the manufacturer's instructions by using the HPV reverse dot blot genotyping kit and the automatic nucleic acid hybridization device (Guangzhou LBP Medical Technology Co., Ltd., Guangzhou, China), which can detect 28 HPV subtypes (6, 11, 16, 18, 26, 31, 33, 35, 39, 40, 42, 43, 44, 45, 51, 52, 53, 54, 56, 58, 59, 61, 66, 68, 73, 81, 82, and 84). We classified HPV types into high risk (HR) types (16, 18, 26, 31, 33, 35, 39, 45, 51, 52, 53, 56, 58, 59, 66, 68, 73, and 82) and low risk (LR) types (6, 11, 40, 42, 43, 44, 54, 61, 81, and 84), based on their association with cervical cancer [18].

### 2.4. Statistical Analysis

A sample size of 79 HIV-infected and 86 HIV-uninfected MSM was required to provide acceptable 95% confidence intervals (CIs) around the expected proportion of men with anal HPV. Median and interquartile range (IQR) were used for continuous variables. Proportions were used for categorical variables. The chi-squared test was used to compare proportions between groups. Rank sum test was used to compare median of anal HPV type detected between groups. Univariate and multivariate logistic regression models were used to calculate odds ratios to estimate the association between potential risk factors and HPV infection. Variables with a *p* < 0.1 using univariate models were entered into the multivariate regression model. Variables with a *p* > 0.1 using univariate models yet deemed as potential confounders were also entered into the multivariate regression model, including age at enrollment, number of male sex partners in the past 3 months, and frequency of condom use in anal sex in the past 3 months. Data were entered using EpiData version 3.0 (The EpiData Association Odense, Denmark) and statistical analyses conducted using STATA version 13.0 (StataCorp, Texas).

## 3. Results

### 3.1. Participant Characteristics

We recruited 79 HIV-infected and 85 HIV-uninfected MSM. As shown in Table 1, HIV-infected MSM and HIV-uninfected MSM were comparable in age (median 26 years for both), ethnicity (96.2% versus 92.9% were Han ethnic), profession (15.2% versus 21.2% were students), marital status (89.9% versus 92.9% were unmarried), and frequency of drinking (*p* for all > 0.1). HIV-infected MSM had slightly less education compared to HIV-uninfected MSM (43.0% versus 60.0% had university education or above, *p* < 0.1). Anal warts were detected in 10.6% of HIV-infected MSM and 3.8% of HIV-uninfected MSM (*p* = 0.095). Sixty-three point three percent of HIV-infected MSM and 30.6% of HIV-uninfected MSM were detected with either anal chlamydia, gonorrhea, or mycoplasma (*p* < 0.001).

### 3.2. Sexual Behaviors

As shown in Table 2, the majority of both HIV-infected (81.0%) and HIV-uninfected MSM (83.5%) had sex with men only. Median age at first anal intercourse (AFAI) with a man (23.7% versus 21.7% with AFAI at 18 years or younger), frequency of condom use in anal sex in the past 3 months (46.8% versus 57.7% always used a condom), sexual experience with a woman in the past 3 months (6.3% versus 8.2%), and commercial sex experience (8.9% versus 4.7%) were similar in both groups of men (*p* for all > 0.1). HIV-infected MSM had slightly less male sex partners in the past 3 months compared to HIV-uninfected MSM, even though the difference was not statistically significant (*p* < 0.1).

### 3.3. Positivity of HPV DNA

All samples were beta-globin positive. The proportion of men with each of the 28 HPV types detected by PCR is shown in Table 3. The positivities of anal HPV of any type (81.0% versus 48.2%, *p* < 0.001), any high risk type (50.6% versus 27.1%, *p* = 0.002), any low risk type (55.7% versus 31.8%, *p* = 0.002), any 4-valent vaccine type (64.6% versus 32.9%, *p* < 0.001), and any 9-valent vaccine type (74.7% versus 36.5%, *p* < 0.001) were all significantly higher among HIV-infected MSM compared to those among HIV-negative MSM. This tendency also applied to HPV 18, HPV 6/11, and HPV 16/18 (*p* for all < 0.05). Other individual HPV types including HPV 6, 33, 39, 52, and 84 were slightly higher among HIV-infected MSM compared to those among HIV-negative MSM, even though the difference was not statistically significant (*p* < 0.1). In the meantime, multiple HPV infection was seen in 30.4% of HIV-infected MSM and 16.4% of HIV-uninfected MSM (*p* = 0.035). HIV-infected MSM had significantly more types of any HPV infection, any 4-valent vaccine type HPV infection, and any 9-valent vaccine type HPV infection compared to HIV-uninfected MSM (median 1 versus 0, *p* for both < 0.05). The great majority of HPV-infected MSM were infected with 9-valent vaccine types (59 out of 64 HIV-infected ones and 31 out of 41 HIV-uninfected ones).

### 3.4. Factors Associated with Anal HPV Positivity

In the multivariate logistic regression model as shown in Table 4, after accounting for other potential confounders: MSM who had an STI detection (including anal chlamydia, anal gonorrhea, or anal mycoplasma) were 2.5 (95% CI: 1.1–6.0) times more likely to be infected with any anal HPV compared to those who had no STI detection; and MSM who had HIV were 4.1 (95% CI: 1.8–9.3) times more likely to be infected with any anal HPV compared to those HIV-uninfected. Rank sum test showed that MSM infected with anal chlamydia (*p* = 0.003) and anal gonorrhea (*p* = 0.058) had greater number of anal HPV types detected.

## 4. Discussion

This was the first study to report anal HPV positivity among MSM in Guangzhou, China. We found, among these predominantly young and sexually experienced MSM, anal HPV positivity in HIV-infected ones was almost twice as high as that in HIV-uninfected ones, and detection of anal chlamydia, gonorrhea, or mycoplasma was associated with both higher anal HPV positivity and greater number of anal HPV types detected.

The rates of anal HPV positivity among both HIV-infected and HIV-uninfected MSM in our study were similar to that reported among MSM in northern and middle China [8–10, 12]. However these rates were higher than that reported among MSM in Shenzhen (71.4% and 33.8%), another large city right next to Guangzhou [13]. The reason might be that men in our study had more sex partners compared to that study (median 2 versus 1.5 in the past 3 months). The Shenzhen study did not recruit HIV-infected and HIV-uninfected MSM separately and only recruited 28 HIV-infected MSM. As a result the point estimate of HPV positivity among HIV-infected MSM might not be reliable.

Many MSM, especially those infected with HIV, had multiple anal HPV types detected. HIV compromises the immunological responses of the body and results in even greater HPV positivity among HIV-infected MSM. Higher reinfection rates and lower clearance rates in HIV-positive MSM may also play a role [19]. The contribution of bacterial infections to HPV infection has not been well documented. Bacterial infections can cause inflammation in the mucosa of the anal canal and understandably facilitate the deposition of HPV in this area. Our study showed that anal bacterial infections, including chlamydia, gonorrhea, and mycoplasma, were associated with both higher anal HPV positivity and multiplicity of anal HPV types. Timely detection and treatment of bacterial infections may help reduce HPV transmission.

The positivities of high risk types including HPV 16, 18, and 52 and low risk types including HPV 6 and 11 were very high in the anal canal in participants, especially in those infected with HIV. The infections of these HPV types suggest high burdens of corresponding morbidities in the foreseeable future, including anal warts that may relapse, anal intraepithelial neoplasia, and even anal cancer. However anal pap smears are not widely available yet in many clinical settings. A review found that only 2 out of 30 national HIV treatment guidelines included anal cancer screening for HIV-infected people [20]. A mathematical modelling demonstrated that screening for anal cancer by incorporating regular anal examinations into routine care for HIV-infected MSM is likely to be cost-effective by conventional standards. Regular anal exams for MSM, especially those living with HIV, to detect anal cancer earlier should be implemented [21]. It is noticeable that the great majority of HPV types detected were covered by both 4-valent and 9-valent HPV vaccines. High immunogenicity and tolerability of both 4-valent and 9-valent HPV vaccines was found in MSM aged 19–26 years. This implied that if vaccinated before sex debut, most of HPV cases in MSM in Guangzhou would be prevented by the two HPV vaccines. However currently in China, the 2-valent HPV vaccine Cervarix manufactured by GlaxoSmithKline was recently approved by the China Food and Drug Administration (CFDA) and this will be the only HPV vaccine that is available in the Chinese market. MSM in China are likely to take this vaccine and actively seek opportunities to get the 4-valent or 9-valent vaccine via other routes. Given that the age at first anal sex has been steadily decreasing [22], many MSM may miss the best age—an age before they commence their sexual life—to be vaccinated against HPV [23]. MSM media and MSM community organizations and clinicians should stand in the forefront of health education about HPV prevention in MSM.

The results in our study should be interpreted with cautions. We used convenience sampling method to recruit participants and our sample may not represent general MSM in Guangzhou. The sample size was comparatively small which limited the statistical power in detecting additional factors associated with HPV positivity. As cross-sectional detection of HPV may be transient deposition only, it was hard to predict how many of the cases would persist. Our study attracted many MSM who experienced receptive anal sex. The majority of men (72%) in our study had receptive anal sex while only 44% had insertive anal sex, in the past 3 months. This may contribute to an overestimated anal HPV infection. Tabaco use had been demonstrated to be an independent risk factor for HPV infection in MSM [24]. We did not ask questions about cigarette smoking and were unable to account for this confounder. Studies showed that smoking rates in both HIV-infected and HIV-uninfected MSM were similar [25]. The missing of this variable was unlikely to cause biased risk for HPV infection when comparing the two groups of men.

Our study found high HPV positivity among MSM in Guangzhou, especially among those infected with HIV. The majority of these cases were potentially preventable by the 9-valent HPV vaccine. Regular anal exams and early HPV vaccination are warranted in MSM attending sexual health clinics in Guangzhou.

## Figures and Tables

**Table 1:** Demographic characteristics among HIV-infected and HIV-uninfected men who have sex with men in Guangzhou.

Demographic characteristic	HIV-infected		HIV-uninfected		All	
	Number/median	IQR/%	Number/median	IQR/%	Number/median	IQR/%
Age at recruitment (year)	26.0	20–38	26.0	20–39	26.0	19–40
18–24	29	36.7	35	41.2	64	39.0
25–34	42	53.2	44	51.8	86	52.4
≥35	8	10.1	6	7.1	14	8.5
Education level^*∗*^						
Middle school or less	19	24.1	13	15.3	32	19.5
Technical diploma	26	32.9	21	24.7	47	28.7
University or above	34	43.0	51	60.0	85	51.8
Ethnicity						
Han	76	96.2	79	92.9	155	94.5
Other	3	3.8	6	7.1	9	5.5
Profession						
Student	12	15.2	18	21.2	30	18.3
Service industry	16	20.3	21	24.7	37	22.6
Private company	15	19.0	10	11.8	25	15.2
Other	36	45.6	36	42.4	72	43.9
Marital status						
Unmarried/divorced	71	89.9	79	92.9	14	8.5
Married	8	10.1	6	7.1	150	91.5
Frequency of drinking						
Every day or often	5	6.3	6	7.1	11	6.7
Sometimes	25	31.7	30	35.3	55	33.5
Seldom or never	49	62.0	49	57.7	98	59.8

*Notes*. ^*∗*^0.05 < *p* < 0.10.

**Table 2:** Sexual behaviors among HIV-infected and HIV-uninfected men who have sex with men in Guangzhou.

Sexual behaviors	HIV-infected		HIV-uninfected		All	
	*n*	%	*n*	%	*N*	%
*Gender of sex partner*						
Mostly with women and sometimes with men	4	5.1	6	7.1	10	6.1
Mostly with men and sometimes with women	11	13.9	8	9.4	19	11.6
With men only	64	81.0	71	83.5	135	82.3
*AFAI (year)*	Median = 19		Median = 19		Median = 19	
≤18	18	23.7	18	21.7	36	22.6
>18	58	76.3	65	78.3	123	77.4
*Type of first sex*						
Mutual masturbation	33	41.8	41	48.2	74	45.1
Oral sex	15	19.0	16	18.8	31	18.9
Anal sex	28	35.4	25	29.4	53	32.3
Cervical sex	3	3.8	3	3.5	6	3.7
*Sex with a man in p3m* ^*∗*^						
No	13	16.5	6	7.1	145	88.4
Yes	66	83.5	79	92.9	19	11.6
*Number of male sex partners in p3m* ^*∗*^	Median = 2		Median = 2		Median = 2	
0	8	11.1	3	3.7	11	7.1
1	32	44.4	50	61.0	82	53.3
≥2	32	44.4	29	35.4	61	39.6
*Frequency of condom use with men in p3m*						
Always	37	46.8	49	57.7	86	52.4
Sometimes	33	41.8	25	29.4	58	35.4
Never	9	11.4	11	12.9	20	12.2
*Sex with a woman in p3m*						
Yes	5	6.3	7	8.2	12	7.3
No	74	93.7	78	91.8	152	92.7
*Commercial sex experience*						
Yes	7	8.9	4	4.7	11	6.7
No	72	91.1	81	95.3	153	93.3

*Notes*. ^*∗*^0.05 < *p* < 0.10.

**Table:** (a) DNA positivity

	*n*, %					
	HIV-infected		HIV-uninfected		All	
	(*N* = 79)		(*N* = 85)		(*N* = 164)	
Any type tested^*∗∗*^	64	81.0	41	48.2	105	64.0
High risk types						
Any high risk types^*∗∗*^	40	50.6	23	27.1	63	38.4
HPV 16	17	21.5	12	14.1	29	17.7
HPV 18^*∗∗*^	8	10.1	2	2.4	10	6.1
HPV 26	2	2.5	1	1.2	3	1.8
HPV 31	2	2.5	0	0	2	1.2
HPV 33^*∗*^	7	8.9	2	2.4	9	5.5
HPV 35	1	1.3	1	1.2	2	1.2
HPV 39^*∗*^	3	3.8	0	0	3	1.8
HPV 45	1	1.3	1	1.2	2	1.2
HPV 51	2	2.5	2	2.4	4	2.4
HPV 52^*∗*^	8	10.1	3	3.5	11	6.7
HPV 53	2	2.5	3	3.5	5	3.1
HPV 56	2	2.5	0	0	2	1.2
HPV 58	6	7.6	2	2.4	8	4.9
HPV 59	5	5.1	2	2.4	6	3.7
HPV 66	2	2.5	2	2.4	4	2.4
HPV 68	5	6.3	3	3.5	8	4.9
HPV 73	0	0	1	1.2	1	0.6
HPV 82	1	1.3	2	2.4	3	1.8
Low risk types						
Any low risk type^*∗∗*^	44	55.7	27	31.8	71	43.3
HPV 6^*∗*^	23	29.1	11	12.9	34	20.7
HPV 11	15	19.0	9	10.6	24	14.6
HPV 40	3	3.8	1	1.2	4	2.4
HPV 42	4	5.1	1	1.2	5	3.1
HPV 43	3	3.8	2	2.4	5	3.1
HPV 44	2	2.5	1	1.2	3	1.8
HPV 54	2	2.5	1	1.2	3	1.8
HPV 61	2	2.5	3	3.5	5	3.1
HPV 81	0	0	2	2.4	2	1.2
HPV 84^*∗*^	0	0	3	3.5	3	1.8
HPV 6/11^*∗∗*^	36	45.6	18	21.2	54	32.9
HPV 16/18^*∗∗*^	25	31.7	14	16.5	39	23.8
Any 4-valent vaccine type^*∗∗*^	51	64.6	28	32.9	79	48.2
HPV 16/18/31/33/45/52/58^*∗∗*^	35	44.3	19	22.4	54	32.9
Any 9-valent vaccine type^*∗∗*^	59	74.7	31	36.5	90	54.9

**Table:** (b) Detection of multiple HPV types

	*n*, %					
	HIV-infected		HIV-uninfected		All	
	(*N* = 79)		(*N* = 85)		(*N* = 164)	
Any type						
Median, IQR^*∗∗*^	1, 0–5		0, 0–4		1, 0–6	
0	15	19.0	44	51.8	59	36.0
1	40	50.6	27	31.8	67	40.9
2	8	10.1	7	8.2	15	9.2
3	6	7.6	3	3.5	9	5.5
4+	10	12.7	4	4.7	14	8.5
4-valent vaccine types						
Median, IQR^*∗∗*^	1, 0–2		0, 0–2		0, 0–2	
0	28	35.4	57	60.1	85	51.8
1	41	51.9	23	27.1	64	39.0
2	8	10.1	4	4.7	12	7.3
3	2	2.5	1	1.2	3	1.8
9-valent vaccine types						
Median, IQR^*∗∗*^	1, 0–3		0, 0–2		1, 0–3	
0	20	25.3	54	63.5	74	45.1
1	41	51.9	23	27.1	64	39.0
2	10	12.7	6	7.1	16	9.8
3	7	8.9	1	1.2	8	4.9
4+	1	1.3	1	1.2	2	1.2

*Notes*. ^*∗*^0.05 < *p* < 0.10. ^*∗∗*^*p* < 0.05.

**Table 4:** Factors associated with detection of any HPV among men who have sex with men in Guangzhou.

Risk factors	% (number of those with HPV/number of men)	Crude odds ratio (95% CI)	Adjusted odds ratio (95% CI)
Age at enrollment			
18–24	57.8 (37/64)	Ref	Ref
25–34	66.3 (57/86)	1.43 (0.74–2.80)	1.45 (0.64–3.29)
≥35	78.6 (11/14)	2.68 (0.68–10.53)	3.07 (0.63–14.99)
Education level			
Middle school or less	78.1 (25/32)	Ref	
Technical diploma	61.7 (29/47)	0.45 (0.16–1.26)	
University or above	60.0 (51/85)	0.42 (0.16–1.08)	
Number of male sex partners in p3m^*∗*^			
0	63.6 (7/11)	Ref	Ref
1	61.0 (50/82)	0.89 (0.24–3.30)	1.51 (0.33–7.04)
≥2	67.2 (41/61)	1.17 (0.31–4.47)	1.17 (0.24–5.72)
Frequency of condom use with male sex partners in p3m			
Always	64.0 (55/86)	Ref	Ref
Sometimes	72.4 (42/58)	1.48 (0.72–3.05)	1.15 (0.50–2.67)
Never	40.0 (8/20)	0.38 (0.14–1.02)	0.41 (0.12–1.40)
Commercial sex experience			
Yes	45.5 (5/11)	0.44 (0.13–1.52)	
No	65.4 (100/153)	Ref	
STI detection^#^			
Yes	74.4 (62/83)	**2.61 **(1.35–5.05)^*∗∗*^	**2.51 **(1.06–5.95)^*∗∗*^
No	53.1 (43/81)	Ref	Ref
HIV status			
Positive	81.0 (64/79)	**4.58 **(2.26–9.27)^*∗∗*^	**4.12 **(1.82–9.32)^*∗∗*^
Negative	48.2 (41/85)	Ref	Ref
Frequency of drinking			
Seldom or never	65.3 (64/98)	Ref	
Sometimes	60.0 (33/55)	0.80 (0.40–1.57)	
Every day or often	72.7 (8/11)	1.42 (0.35–5.69)	

*Notes*. ^*∗∗*^*p* < 0.05. ^#^STIs include anal chlamydia, anal gonorrhea, and anal mycoplasma. ^*∗*^0.05 < *p* < 0.10.
